# The Effect of Nano-Biochar Derived from Olive Waste on the Thermal and Mechanical Properties of Epoxy Composites

**DOI:** 10.3390/polym17101337

**Published:** 2025-05-14

**Authors:** Muhammed İhsan Özgün, Vildan Erci, Emrah Madenci, Fatih Erci

**Affiliations:** 1 Department of Metallurgy and Material Engineering, Faculty of Engineering, Necmettin Erbakan University, Konya 42090, Türkiye; miozgun@erbakan.edu.tr; 2 Department of Soil Science and Nutrition, Faculty of Agriculture, Selcuk University, Konya 42130, Türkiye; vildanerci@selcuk.edu.tr; 3 Department of Civil Engineering, Necmettin Erbakan University, Konya 42090, Türkiye; 4 Science and Technology Research and Application Center (BITAM), Necmettin Erbakan University, Konya 42090, Türkiye; 5 Department of Biotechnology, Faculty of Science, Necmettin Erbakan University, Konya 42090, Türkiye

**Keywords:** nano-biochar, epoxy composite, green filler, mechanical properties, thermal stability, sustainable materials, olive pulp

## Abstract

The increasing demand for the development of environmentally friendly alternatives to petroleum-derived materials has increased research efforts on sustainable polymer composites. This study systematically examined the effect of nano-biochar derived from agricultural wastes such as olive pulp on the mechanical and thermal properties of epoxy-resin-based composites. First, the biochar from olive pulp was produced by pyrolysis at 450 °C and turned to nano-biochar using ball milling. Composite samples containing nano-biochar at different rates between 0 and 10% were prepared. The nano-biochar and composite samples were characterized by using different techniques such as SEM-EDS, BET, FTIR, XRD, Raman, TGA, and DMA analyses. Also, the tensile strength, elastic modulus, Shore D hardness, thermal stability, and static toughness of the composite samples were evaluated. The best performance was observed in the sample containing 6% nano-biochar; the ultimate tensile strength increased from 17.37 MPa to 23.46 MPa compared to pure epoxy, and the elastic modulus and hardness increased. However, a decrease in brittleness and toughness was observed at higher additive rates. FTIR and DMA analyses indicated that the nano-biochar interacted strongly with the epoxy matrix and increased its thermal stability. The results showed that the olive-pulp-derived nano-biochar could be used to improve the structural and thermal properties of the epoxy composites as an inexpensive and environmentally friendly filler. As a result, this study contributes to the production of new polymer-based materials that will encourage the production of environmentally friendly composites with nano-scale biochar obtained from olive waste, which is an easily accessible, renewable by-product.

## 1. Introduction

The search for new eco-sustainable and bio-based composites has attracted much attention, especially concerning environmentally friendly systems derived from waste and renewable resources [1]. In this respect, a promising alternative to traditional carbonaceous fillers is biochar (BC), a carbonaceous and renewable material produced by the thermochemical transformation of biomass in an oxygen-limited environment [2]. Unlike other carbon-based materials, BC is derived from sustainable biomass sources and has high thermal stability and hardness, high surface area, good chemical stability, and electrical conductivity [3,4].

Polymer matrix composites are one of the advanced materials that have received the most research due to their wide variety of uses in various critical industrial products, ranging from the aerospace to the automobile industry [5,6,7]. These applications are often based on materials reinforced with carbon black, glass/carbon fibers, and carbon nanotubes (CNTs) [8,9]. Carbon fillers are generally incorporated into polymers to improve their mechanical, thermal, electrical, and chemical corrosion resistance properties compared to metal-filled composites. These materials can be used to achieve excellent composite performance [10]. The production of petroleum-based carbon materials requires laborious synthetic methods and is environmentally and economically unviable. Efforts have been made to explore various renewable sources of carbon as raw materials that are environmentally friendly, cost-effective, and abundant in nature.

In this regard, the number of studies addressing the formulation of BC-containing polymeric systems based either on thermoplastic or thermosetting matrices has been expanding exponentially over the last several years [11]. BC is an interesting alternative to standard carbonaceous fillers for the purpose of increasing the mechanical, electrical, and physical properties of polymer-based composites [12]. This situation is explained by BC’s exceptional qualities, capacity for functionalization, and structural flexibility [13,14]. Additionally, research has established that the physical bonding resulting from the infiltration of polymer chains into the BC pores is crucial for achieving composite materials with enhanced mechanical properties, thereby negating the necessity for coupling agents to improve interfacial adhesion between the BC particles and the polymer matrix [15]. Also, BC is more advantageous than natural fibers as a filler in polymer composites because its properties can be modified by changing the pyrolysis conditions to achieve a hydrophobic structure in BC and more excellent compatibility with the polymer matrix [16]. It has been reported that the thermal stability of the obtained BC composites is higher than that of composites with natural fibers [17].

BC is a carbon-rich material obtained from various agricultural and forestry carbon-containing solid wastes produced by pyrolysis. In the past, a large number of studies on the application of BC from waste have mainly focused on soil enrichment, pollutant removal, and carbon sequestration due to its low cost, high carbon content, and adsorption capacity [18]. However, recent research has highlighted BC’s potential application in polymer matrix composites due to the similar properties of carbon black and carbon nanotubes [19,20,21]. In their first comparative and thorough investigation, Khan and Savi [22] evaluated the mechanical performance of epoxy composites containing varying concentrations of CNTs and maple-derived BC. They achieved this by conducting tensile tests on the composites. Compared to the insertion of CNTs, implementing a BC concentration that did not exceed 2 wt.% resulted in an improvement in the overall mechanical performance of the epoxy-based composites. In spite of this, a greater filler content was necessary in order to obtain the same electrical conductivity as that obtained when using CNTs. Compared to the composites that included 4 wt.% of carbon nanotubes, the conductivity of a 20 wt.% BC formed at 900 °C was found to be greater in the AC mode. An epoxy matrix was filled with 15 wt.% of coffee-derived BC by Giorcelli and Bartoli [23], who measured the conductivity of the material to be 36 S/m. In spite of the fact that the high filler loading resulted in a 47% reduction in the elongation at break and a doubling of Young’s modulus, this was much greater than that achieved by employing the same quantity of carbon black. The matrix’s restricted mobility, which prevented it from effectively redistributing the stresses that were applied, was the cause of the increase in brittleness that occurred. This is also connected to the thermal stage and ramps used during the synthesis of BC, which is tied to the performance of epoxy composites that include BC. According to the findings of Bartoli and Nasir [24], the highest temperature attained during the carbonization of olive trunks is an essential parameter for the purpose of modifying the mechanical properties of the composites that are finally produced. The authors investigated epoxy composites that included two weight percent of BC. These composites were created at four distinct temperatures (namely, 400 °C, 600 °C, 800 °C, and 1000 °C) and three different heating speeds (5 °C, 10 °C, and 50 °C per minute). BC filler derived from waste pine wood was included in a polypropylene matrix using melt extrusion at several loadings (6 to 30 wt.%) and at two distinct pyrolysis temperatures of 400 and 450 °C, alongside 30 wt.% of wood by Das and Sarmah [25]. The mechanical analysis of the composites indicated that the material with 24 wt.% of BC had a comparable tensile strength and modulus; however, it had superior flexural capabilities relative to standard wood/PP composites. The results were ascribed to the enhanced interfacial adhesion between the wood and polypropylene due to the inclusion of bacterial cellulose. Nonetheless, a significant decrease in material ductility was seen for BC loadings over 15 wt.%. Giorcelli et al. [26] reported a morphological characterization of BC and heat-treated BC in order to evaluate the differences. The BCs were then dispersed in an epoxy resin to produce composites. The BC and heat-treated BC were pyrolyzed in an inert atmosphere at 600 °C and 1000 °C, respectively. The best results were obtained by adding both BC concentrations at 1% by weight.

The pyrolysis process produces an appropriate biochar with a proper processing temperature [27]. Due to temperature variations, such as a lower pyrolysis temperature, materials with superior mechanical properties and volatile compounds are obtained at maximum levels during the heating process. The pyrolysis temperature and raw materials are essential aspects of the main process parameters for improving biochar’s qualities.

As stated in the literature, this carbon-based material, which is low-cost and environmentally friendly, has uses in different areas, and biochar could become the leader among carbon materials in composites. BC has been widely researched for environmental remediation, catalyst support, and energy storage applications. However, BC-based composites still require optimization to achieve comparable performance to traditional carbon-based fillers such as graphene and carbon nanotubes [28].

Despite the growing interest in the literature for the use of BC as a sustainable carbon-based filler material, the effects of BC on the mechanical properties of epoxy-based composites have not been adequately investigated, especially for nano-scale BC obtained from olive waste. Although previous studies have shown that BC has the potential to increase thermal stability and electrical conductivity in polymer matrices, extensive research on its mechanical strengthening capabilities is limited. Furthermore, the relationship between the BC content, dispersion quality, and mechanical performance remains not fully elucidated. This study systematically investigated the effects of nano-BC filler on the mechanical performance of epoxy resin composites, and a comprehensive characterization process was applied. While the direct effects of the nano-BC additive on the tensile strength, modulus of elasticity, and toughness properties were determined, thermal stability, chemical structure, and microstructure analyses were carried out at the same time. In this study, samples with different percentages (0 wt.%, 2 wt.%, 4 wt.%, 6 wt.%, 8 wt.%, 10 wt.%) of nano-BC content by weight were produced, and the mechanical, thermal, and structural properties of these samples were evaluated in detail. In particular, an important point that distinguishes this study from similar studies in the literature is the use of olive pomace in the production of the nano-BC. Olive pomace is a byproduct of the food and agriculture industry, a source of biomass that is often treated as waste but that is rich in carbon. In this study, the effect of converting olive pomace to nano-scale BC by controlling the pyrolysis process and using it as a filling material in epoxy composites on their mechanical and thermal properties was examined in detail. Thus, it was ensured that industrial waste was transformed into a high-value-added material, and a sustainable alternative to traditional carbon-based filling materials is offered.

## 2. Materials and Methods

**Figure 1:**
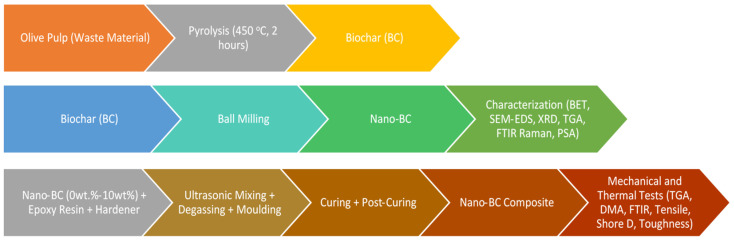
Flowchart of study.

In this study, the mechanical and thermal properties of epoxy-based composites were investigated using nano-BC obtained from olive pulp. This study was carried out in three main stages: (1) nano-BC production, (2) characterization studies, and (3) composite production and testing processes. A general flow chart of this process is presented below (Figure 1).

### 2.1. Production of the Nano-BC via Ball Milling

**Figure 2:**
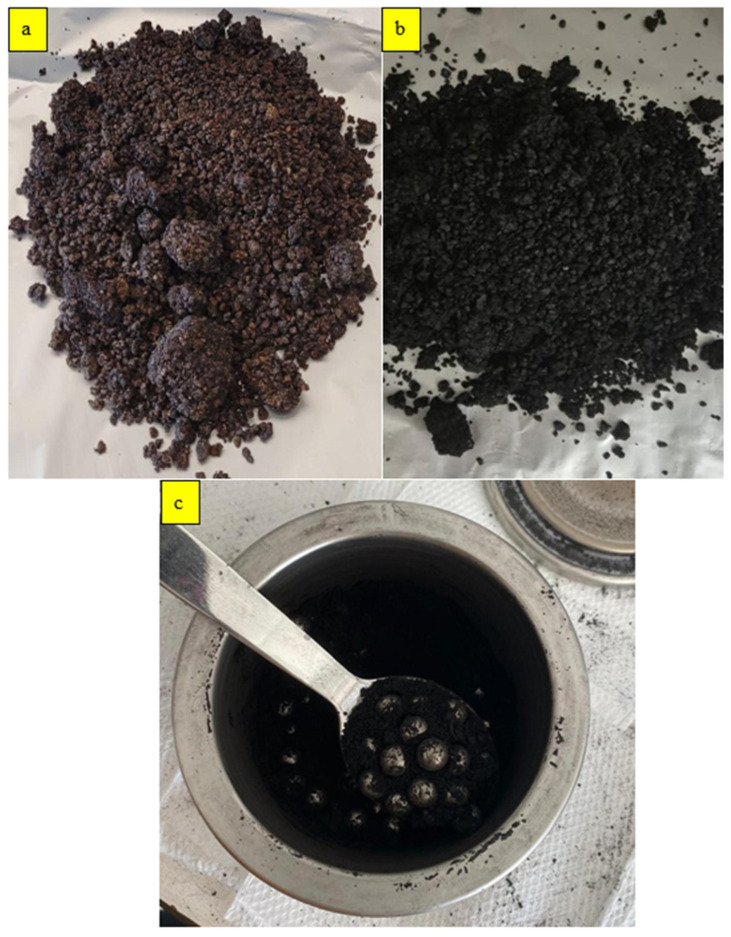
Process of nano-BCs: (**a**) olive pomace; (**b**) BC; (**c**) ball milling.

This study utilized olive pomace, a by-product of olive oil production, to produce BC. In the analysis of the olive pomace feedstock, the sample–water mixture at a 1:1 ratio exhibited a pH of 4.92 and an electrical conductivity (EC) of 0.547 S/m. Additionally, the moisture content of the sample was determined to be 57.08%. The pyrolysis process was conducted at a temperature of 450 °C. The pyrolysis was carried out for 2 h at a heating rate of 10 °C/min (Figure 2a,b). Following pyrolysis, the resulting BC was stored for further processing to reduce its size to the nano scale. The BCs were converted into nano-BCs using a planetary ball mill. In this process, 500 mL hardened steel jars were filled with steel balls in a 1:10 weight ratio to BC (Figure 2c). The milling process was carried out at a speed of 400 rpm in 15 min periods for 30 min, during which the biochar was subjected to mechanical forces that significantly reduced its particle size. To prevent excessive heating and degradation of the biochar during milling, the process was intermittently stopped and restarted at different time intervals.

### 2.2. Characterization of the Nano-BC

The specific surface area of the nano-biochar was determined using the Brunauer–Emmett–Teller (BET) method, which involved analyzing nitrogen adsorption isotherms at a temperature of 77.3 K, revealing important insights into the porosity and surface characteristics of the biochar [29]. The nano-BC was degassed for 24 h at 80 °C under vacuum (10^−2^ mbar) before BET analysis. Scanning Electron Microscopy (SEM) was employed to investigate the nano-BC’s size and morphological characteristics. Additionally, the mineral phases present in the samples were identified using Energy Dispersive Spectroscopy (EDS) in conjunction with SEM under controlled conditions of the acceleration voltage (20 kV) and beam current. However, the nano-BC’s particle size distribution was analyzed by a nano-particle sizer. Thermogravimetric analysis (TGA) was conducted to evaluate the nano-BC’s structural stability, allowing for the observation of weight loss patterns under regulated atmospheric conditions, which provided insights into the material’s thermal stability. A total of 20 mg of the material was heated at a rate of 10 °C/min from 25 to 900 °C in a nitrogen environment. To determine the crystalline phases of the nano-BC, X-ray diffraction (XRD) examination was performed in the 2θ range of 5–90° using Cu Kα radiation (λ = 1.5406 Å). Using a 532 nm laser (Renishaw, inVia Reflex, Wotton -under-Edge, UK), Raman scanning was carried out between 125 and 4000 cm^−1^ wavelengths in addition to XRD examination to identify the carbon allotrope of the nano-BC structure. Fourier-transform infrared (FTIR) spectroscopy was employed to investigate the chemical functional groups present in the nano-BC, aiding in understanding its reactivity and interaction with other substances. FTIR spectra were taken between 400–4000 cm^−1^ wavelengths.

### 2.3. Preparation of the Nano-BC Composite Samples

**Figure 3:**
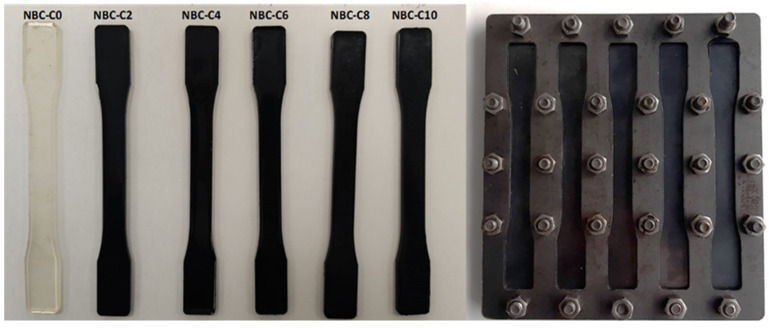
Dog-bone composite samples and mold.

According to the recipe shown in Table 1, composite samples were obtained by adding nano-biochar (nano-BC) to epoxy resin. Within the scope of this study, six types of composite samples containing nano-biochar in different proportions were produced. The NBC-C0 sample contained only pure epoxy resin, the NBC-C2 sample contained 2% nano-biochar, the NBC-C4 sample contained 4% nano-biochar, the NBC-C6 sample contained 6% nano-biochar, the NBC-C8 sample contained 8% nano-biochar, and the NBC-C10 sample contained 10% nano-biochar supplement. LR350-coded resin and LG350-coded hardener supplied from Dost Kimya A.Ş. (Istanbul, Türkiye) were used as the epoxy resin. The epoxy resin used in this study had a 600–900 mPa·s viscosity and was a two-phase mixture comprising 80–90% diglycidyl ether bisphenol A and 10–20% aliphatic diglycidyl ether. In order to obtain an optimal dispersion of the fillers in the matrix, a mixture of epoxy resin and nano-BC was used by immersing a TT13 ultrasonic probe (SONOPLUS HD2200, Bandelin, Berlin, Germany) for 5 min. The ultrasonic probe homogenization was performed in an ice bath at 0 °C. Gas bubbles trapped in the fluid mixture in the glass beaker were removed by sonication (Elma sonic P60H, Singen, Germany) for 10 min. The mixture was then poured into a metal mold made according to ASTM D 638-4 [30] to produce five specimens in the shape of dog bones. The dimensional tolerance of this mold was ±0.1 mm for all specimens. Finally, degassing was carried out in a low vacuum chamber (50 mbar) for 20 min. Five samples of each concentration were prepared to monitor reproducibility and subject the results to statistical analysis (Figure 3). The curing process was carried out at room temperature (25 °C) for 24 h. Subsequently, the samples were post-cured at 80 °C for 6 h. The curing and post-curing parameters were based on the resin’s recipe for the user. However, in the post-curing process, there was no significant increase in the Shore D hardness value, which was determined as 6 h, and after post-curing at a longer time and higher temperature, warping and demolding problems were encountered in the samples. The amount of the mixture combination of nano-BC, epoxy resin, and hardener was determined as 75 g for five samples, and the distribution according to the amount of nano-BC is given in Table 1. During the process, a catalyst and accelerator were employed to ensure a more uniform dispersion of the two materials.

TGA analysis was performed to determine the thermal behavior of the composite samples produced by doping nano-BC with pure epoxy. TGA analysis was conducted in a nitrogen atmosphere with a heating rate of 10 °C/min from room temperature to 900 °C. From the TGA analysis, the effect of the nano-BC additive amount on the thermal strength was investigated. Dynamic mechanical analysis (DMA) samples with dimensions of 2 mm × 10 mm × 20 mm were produced from composite structures produced with pure epoxy and 2, 4, 6, 8, and 10 wt.% nano-BC additives. DMA analysis was performed in the single cantilever mode at a frequency of 1 Hz at a heating rate of 3 °C/min up to 60 °C starting from −20 °C. Since the maximum application temperature of the resin is 50 °C, temperatures up to 60 °C were used. Fourier-transform infrared (FTIR) spectroscopy was used to investigate the interaction of the pure epoxy and nano-BC doping at the molecular level. In addition to these analysis and test methods, the Shore D hardness values of the pure epoxy samples and nano-BC-doped composites were measured according to ASTM D2240 [31].

## 3. Results and Discussion

### 3.1. Brunauer–Emmett–Teller (BET) Analysis

**Figure 4:**
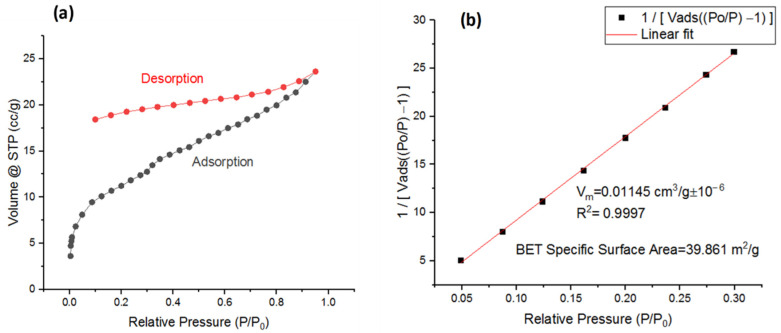
(**a**) Nano-BC adsorption–desorption isotherm; (**b**) specific surface area calculation of nano-BC.

The properties of the surface area and porosity of the nano-BC were evaluated from nitrogen adsorption isotherms at 77.3 K using the Brunauer–Emmett–Teller (BET) method. The adsorption–desorption curve obtained a Type IVa isotherm according to the IUPAC classification (Figure 4a). This is characteristic of mesoporous materials and demonstrates a strong correlation between the pore volume and surface area of the material [32]. The specific surface area of the nano-BC was calculated as 39.861 m^2^/g (Figure 4b). This reveals that the biochars obtained at the nano scale had a very high surface area and offered high interfacial interaction potential within the epoxy matrix. Thanks to this structure, polymer chains could physically penetrate the porous structure of the nano-BC, which could create interfacial interactions that provide effective load transfer without the need for a coupling agent.

Similarly, Giorcelli and Bartoli [23] reported that BC from coffee waste provides effective bonding with an epoxy matrix due to its high specific surface area and mesoporous structure. In a relevant study, a 2.02 S/m electrical conductivity was obtained at a 15% filling ratio; however, in response to this increase, the ultimate elongation decreased from 3.50% to 1.16%; thus, it was observed that the composites became brittle. This comparison reveals that nano-BC has the potential to form mechanical bonds due to its high surface area; however, in case of excessive doping, a loss of ductility and increase in brittleness should also be taken into consideration.

### 3.2. SEM-EDS Analysis of the Nano-BC

**Figure 5:**
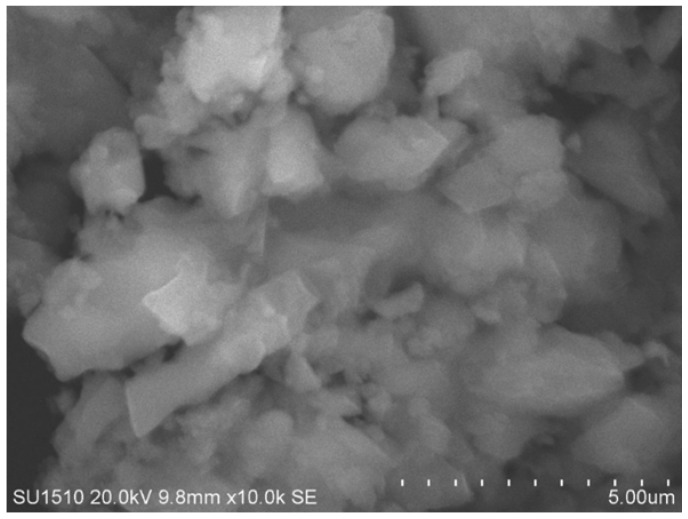
SEM micrograph of nano-BC particles.

The morphological properties of the nano-BC obtained from olive pulp were examined by Scanning Electron Microscopy (SEM), and it was observed in the images obtained that the particles had a graphitic layered structure and were of a submicron size (Figure 5). This structure contributed to the efficient load transfer by providing a high surface contact area in the epoxy matrix. The layered graphitic structure increased the physical interaction level of the nano-BC with the matrix; this can be directly associated with increases in properties such as tensile strength and hardness.

**Figure 6:**
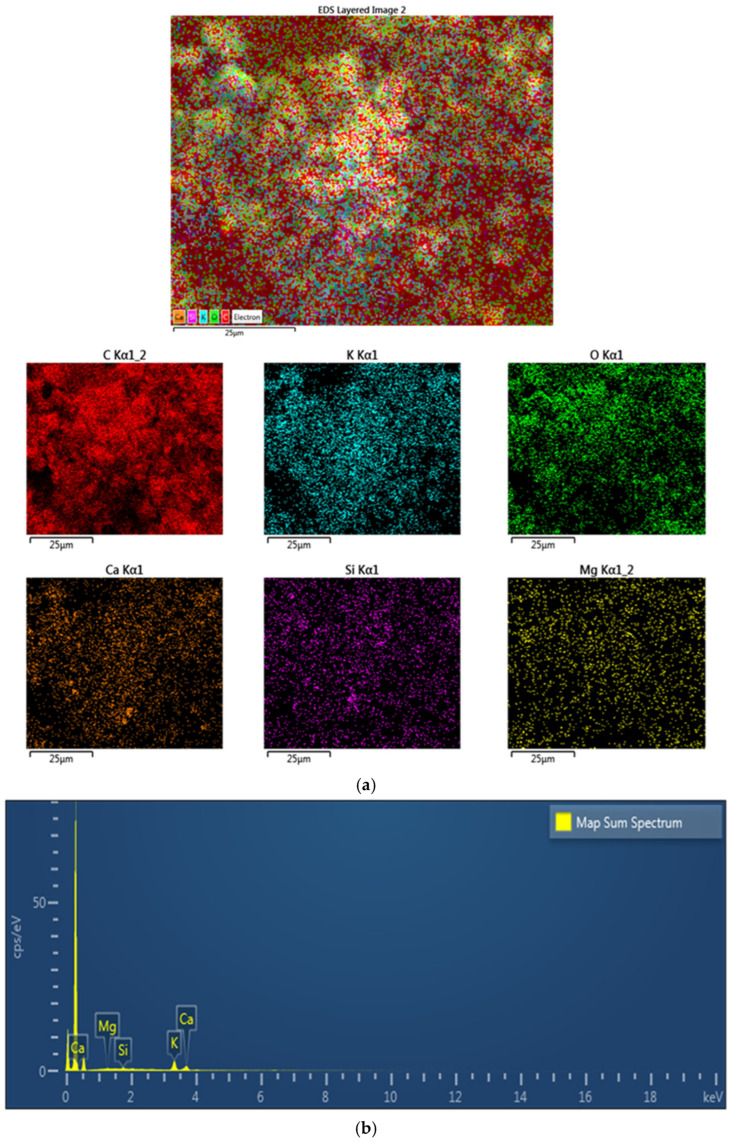
(**a**) SEM micrograph of nano-BC particles and EDS elemental mapping of nano-BC particles, (**b**) EDS elemental spectrum of nano-BC particles.

The elemental composition of the nano-BC obtained by Energy Dispersive Spectroscopy (EDS) mapping analysis is presented in Figure 6. Accordingly, the density of carbon (C) and oxygen (O) elements was high; trace elements such as calcium (Ca), potassium (K), silicon (Si), and magnesium (Mg) were also detected. The weight percentages of the relevant elements are given in Table 2. As a result of the EDS analysis, the basic composition of the nano-BC was determined as C (81.23%), O (16.36%), K (1.50%), Ca (0.72%), Si (0.12%), and Mg (0.08%) (Table 2). The high carbon content indicates the effectiveness of the pyrolysis process; oxygen and other minerals indicate that the source-specific inorganic structures were partially preserved. These minerals created microhardness and indirect bonding effects in the epoxy matrix. This elemental structure indicates that the bond established with the polymer matrix could be supported not only physically but also chemically. Oxygen-containing surface groups could increase the strong adhesion at the interface through hydrogen bonds or dipole–dipole interactions with epoxy chains.

### 3.3. Particle Size Distribution of the Nano-BC

**Figure 7:**
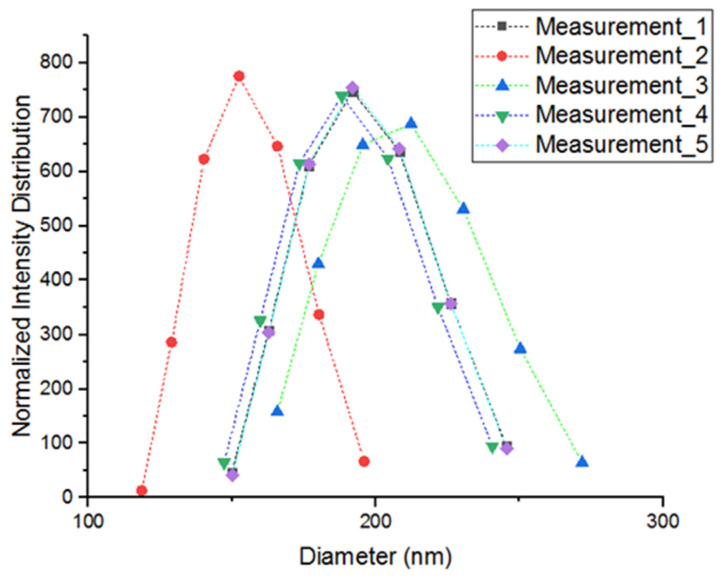
Nano-BC particle size distribution.

The particle size distribution of the nano-BC was determined by the dynamic light scattering (DLS) method using a NanoPlus HD-3 (Norcross, GA, USA) instrument (Figure 7). Figure 7 clearly reveals that the size distribution was narrow and unimodal. Measurements were carried out on ultrasonically dispersed samples in a pure water medium at room temperature.

The data obtained are summarized in Table 3. The average particle diameter of the nano-BC was measured as 225.5 nm, and the polydispersity index (PDI) was found to be 0.183. A PDI below 0.2 indicates that the system is monodisperse or narrowly distributed and has a low tendency to agglomerate. This situation reveals both that the milling process was successful and that the dispersion process was effective in preventing particle-to-particle interactions. In this size range (below 300 nm), the particles provided a larger interface area within the polymer matrix, increasing the effectiveness of load transfer and bonding mechanisms. In addition, the homogeneity of the size distribution prevented irregularities that create stress concentrations within the matrix, positively affecting the mechanical performance of the structure. The nano-BC obtained in this study demonstrated that it could provide effective interfacial interaction in epoxy resin systems due to its fine size and low PDI.

### 3.4. Thermogravimetric Analysis (TGA)

Thermogravimetric analysis (TGA) was used to measure the resistance temperatures of the nano-BC and nano-BC–epoxy blend composites against thermal degradation and to determine the degradation mechanisms. As a function of temperature, the sample’s weight loss was documented.

**Figure 8:**
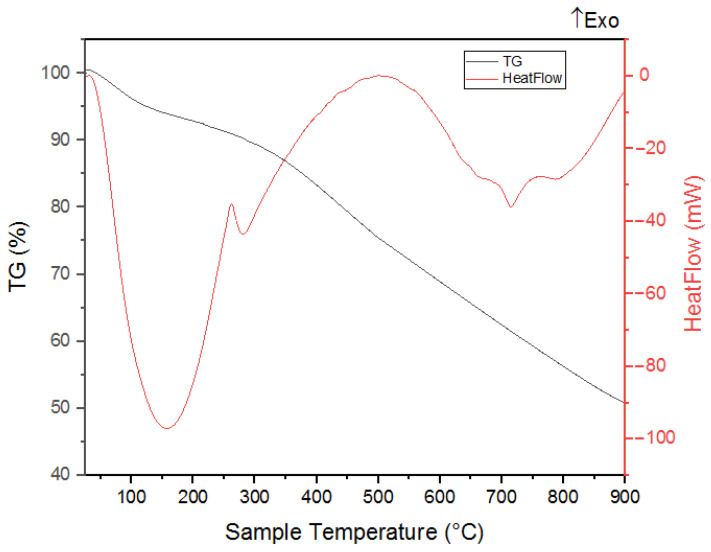
TGA and DSC (heat flow) analysis of nano-BC.

The nano-BC’s TGA and DSC graph in Figure 8 reveals that it contained about 5% moisture. However, volatile compounds and organic structures were present in the nano-BC, as seen from the endothermic DSC peak at 200–600 °C. This presence showed around 20% volatile matter in the structure, except for free moisture, between 200–600 °C, as shown in Figure 8. Following the temperature increase beyond 650 °C, the material exhibited an endothermic reaction, indicating the onset of thermal decomposition.

**Figure 9:**
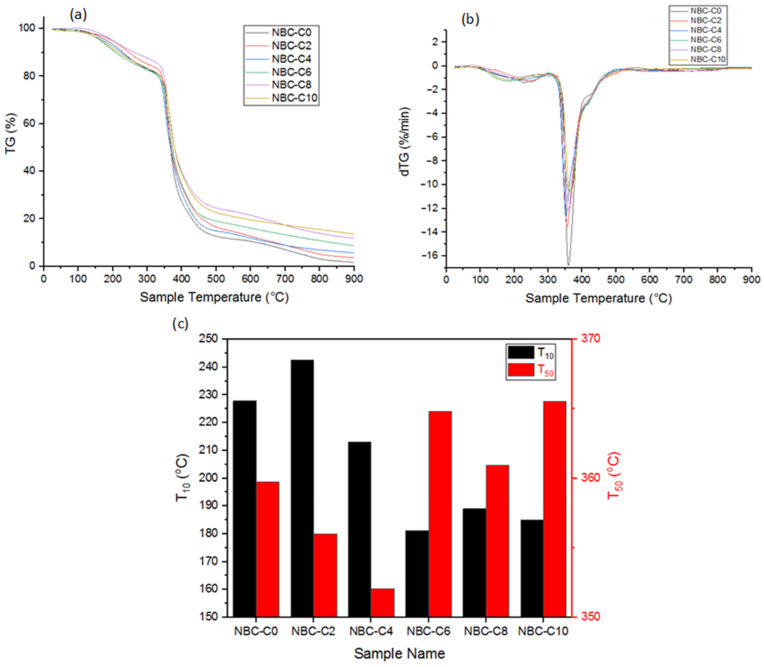
(**a**) TGA analysis of composites; (**b**) dTG of composites; (**c**) temperatures of composites for T_10_ and T_50_.

Figure 9a shows the TGA graphs of the epoxy control sample and nano-BC-doped composites. The effect of the TGA graph of the epoxy resin on the thermal decomposition temperature of the composite samples prepared by doping at different ratios was limited. Figure 9b,c shows the variation in dTG, T_10_ (temperature at 10% mass loss, i.e., temperature at which thermal degradation is considered to start), and T_50_ (temperature at 50% mass loss, i.e., temperature at which thermal degradation is considered to be maximum) according to the nano-BC additive ratios. Adding graphitic nano-BC at different ratios to the epoxy had two distinct effects on its thermal stability. Increasing the nano-BC doping facilitated the removal of volatile molecules from the epoxy matrix (decrease in T10 temperature) but increased the fragmentation temperature of the main chain (increase in T50 temperature). Although the increase in the temperature value required for complete thermal degradation was limited with nano-BC doping, the degradation rate slowed down, as can be seen in the dTG graph (Figure 9b).

### 3.5. Fourier-Transform Infrared Spectroscopy (FTIR)

**Figure 10:**
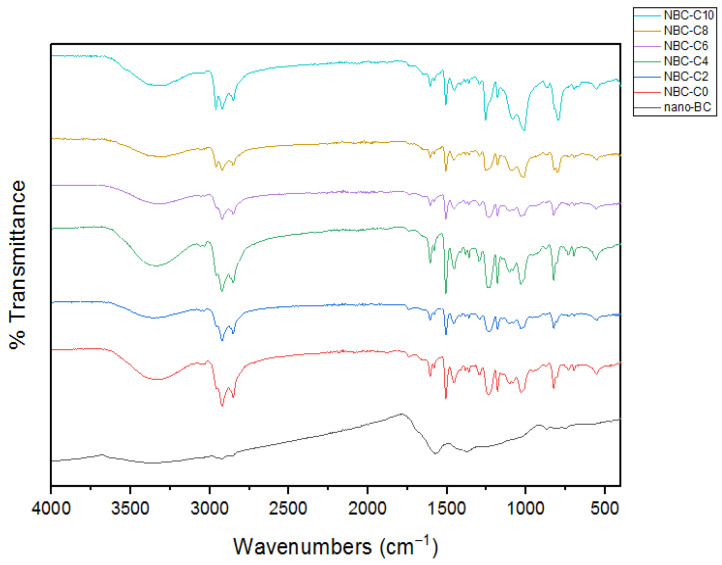
Characterization of the neat epoxy, nano-BC, and its composites: FTIR.

Fourier-transform infrared spectroscopy (FTIR) analysis was conducted to examine the chemical composition and functional groups of the pure epoxy resin, nano-BC, and nano-BC-doped epoxy composites with different weights (wt.%) of nano-BC. Figure 10 shows the FTIR spectra of the neat epoxy, nano-BC, and nano-BC composites. In the obtained spectra, the broad band observed in the ~3300 cm^−1^ region corresponds to the hydroxyl (-OH) groups in the epoxy resin, and significant changes occurred in this band with the increase in the BC content. The C-H stress bands located in the ~2900 cm^−1^ region represent the aliphatic groups of the epoxy matrix. Furthermore, a significant increase in aromatic C=C vibration bands in the ~1500–1600 cm^−1^ region was observed as the BC content increased; this suggests that the BC interacted with the epoxy matrix, causing structural changes. Slight shifts and density changes also occurred in the C-O-C vibration bands in the range of ~1200–1000 cm^−1^, indicating the presence of epoxy rings. These changes suggest that the BC was incorporated into the epoxy matrix, entered chemical interactions, and affected the material’s structural integrity.

### 3.6. X-Ray Diffraction (XRD)

**Figure 11:**
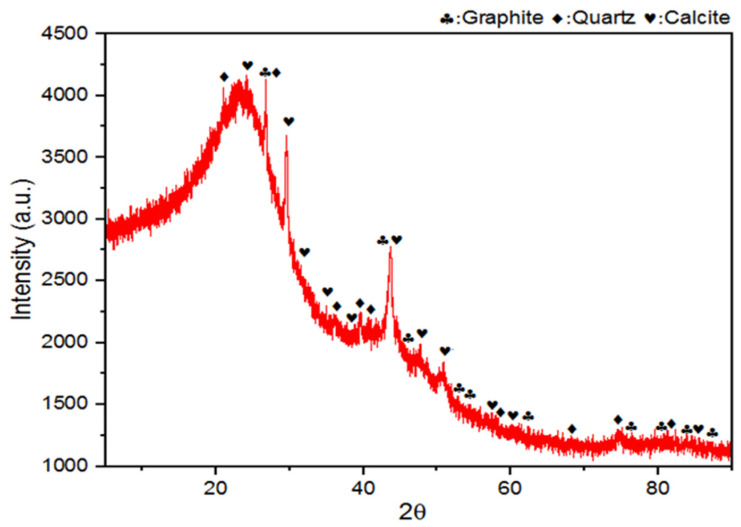
XRD spectrum of nano-BC particles.

X-ray diffraction (XRD) analysis was performed on the nano-BC particles, and the spectrum given in Figure 11 was obtained. The phases matched through the HighScore Plus© version 4.6a program were graphite (ICSD code: 29123), calcite (ICSD code: 18164), and quartz (ICSD code: 166601). The endothermic DSC peak and mass decrease after 650 °C in the TGS analysis can be confirmed by the calcination of CaCO_3_. The stacking of aromatic graphite layers between 20° and 30° is represented by the diffraction features in the XRD spectrum that the nano-BC displayed. The existence of tiny crystallites orientated perpendicular to these aromatic layers was responsible for the region’s broadening [33].

### 3.7. Raman Spectroscopy Analysis

**Figure 12:**
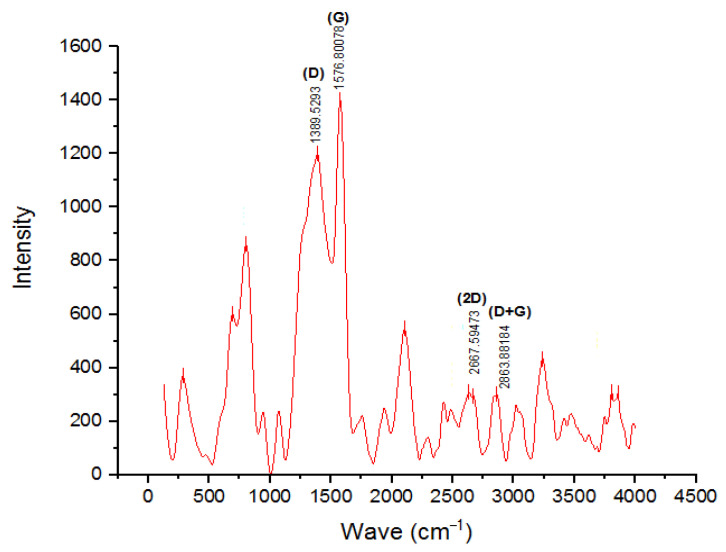
Raman pattern of nano-BC particles.

The Raman spectrum of the nano-BC sample is shown in Figure 12. Raman spectroscopy was also utilized in addition to XRD examination to ascertain whether or not the nano-BC structure was in a graphite structure. The nano-BC characteristically had a D band around 1350 cm^−1^ due to structural defects and a G band around 1590 cm^−1^ due to sp^2^ hybridization. The intensity of the peaks in these Raman shift regions indicates that the structure was an amorphous or graphite structure. Since the D/G ratio was less than 1, it was determined that the structure was graphitic. The presence of prominent peaks of the 2D band around 2700 cm^−1^ and the D+G band around 2900 cm^−1^ in the Raman pattern indicates that the structure was a graphite structure showing sp2 hybridization [34].

### 3.8. Characterization of the Nano-BC Using DMA Analysis

**Figure 13:**
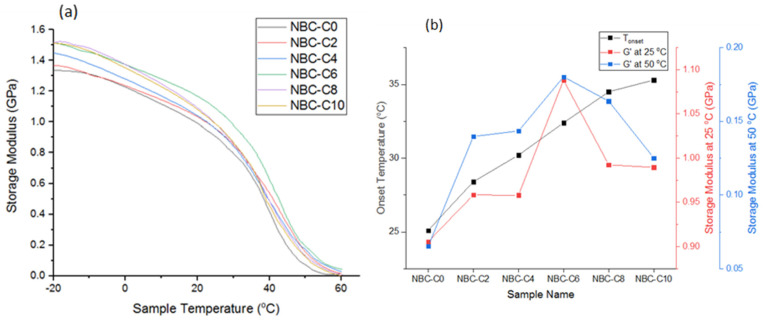
(**a**) Variation in storage modulus with temperature of composites; (**b**) onset temperature and storage modulus at 25 °C and 50 °C of composites.

The thermomechanical effects of the nano-BC additive on the epoxy matrix were evaluated by dynamic mechanical analysis (DMA). As a result of the measurements, it was observed that the sample with a 6% additive ratio (NBC-C6) exhibited the highest storage modulus (E′) at both 25 °C and 50 °C. This shows that the nano-BC acted as a reinforcing phase that limited the movement between the polymer chains and provided effective load transfer at the interface. The high surface area and graphitic hardness of the nano-BC supported the mechanical stability enhancing effect of this additive. The DMA results show that the additive ratio was critical in optimizing the behavior of the epoxy matrix against temperature. The 6% additive ratio represents the optimum level in terms of both hardness and elastic response. These findings clearly demonstrate the effect of the nano-BC, when used at appropriate concentrations in the matrix, on increasing the strength of the structure against temperature. Figure 13a presents the storage modulus change; Figure 13b presents the onset temperature (Tonset) and storage modulus at 25 °C and 50 °C of the composites values in a comparative manner. The softening Tonset increased up to an additive content of 6% and showed a decreasing trend with the further increase in the additive ratio. This situation shows that while the nano-BC provided thermal resistance at low additive ratios, it created deteriorations due to not being homogeneously distributed in the matrix at high additive ratios.

### 3.9. Hardness Test of the Composites

**Figure 14:**
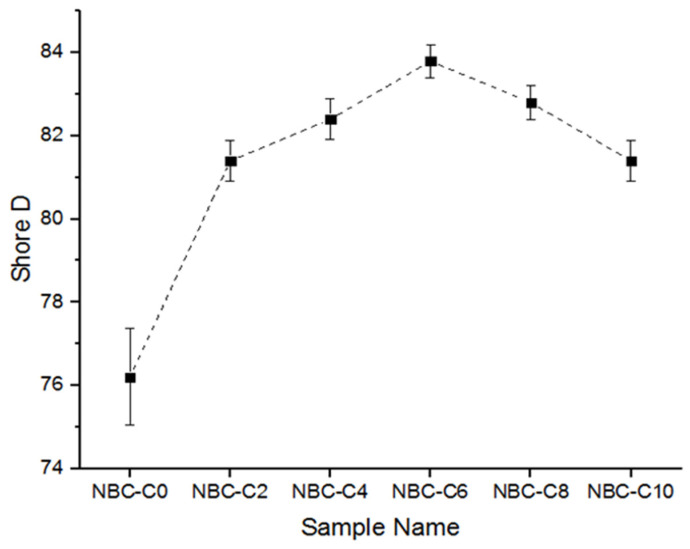
The Shore D values of the composites.

The effect of the nano-BC additive on the surface hardness of the epoxy resin was evaluated by the Shore D method. The measurements were taken from five replicate samples for each additive ratio, and the average values were calculated, and the results are presented in Figure 14. According to the results obtained, an increase in hardness was observed starting from a 2% additive ratio, and the highest hardness was obtained at a 6% additive ratio. This was due to the effective dispersion of the nano-BC in the matrix and its hard structure acting as a phase that increased the mechanical resistance.

This increase in the Shore D hardness value can be attributed to the resistance of the nano-BC to the micro-level deformation of the matrix due to its high carbon content and porous structure. In particular, the increase up to a 6% contribution rate shows that the nano-filler provided reinforcement to the structure. However, when the contribution rate reached the 8% and 10% levels, the increasing trend in hardness values was halted or a limited decrease was observed. This situation shows that the nano-BC tended to agglomerate in the matrix at high contribution rates, thus disrupting the homogeneous distribution and creating local weak points. These results reveal that the nano-BC additive had a direct effect not only on the strength but also on the surface resistance; however, the necessity for careful optimization of the additive ratio should be emphasized.

### 3.10. Ultimate Tensile Strength of the Nano-BC Composites

**Figure 15:**
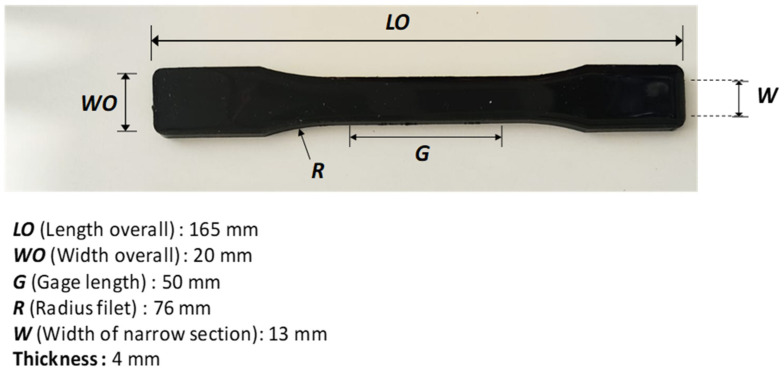
Geometric dimensions of a dog-bone-shaped sample.

**Figure 16:**
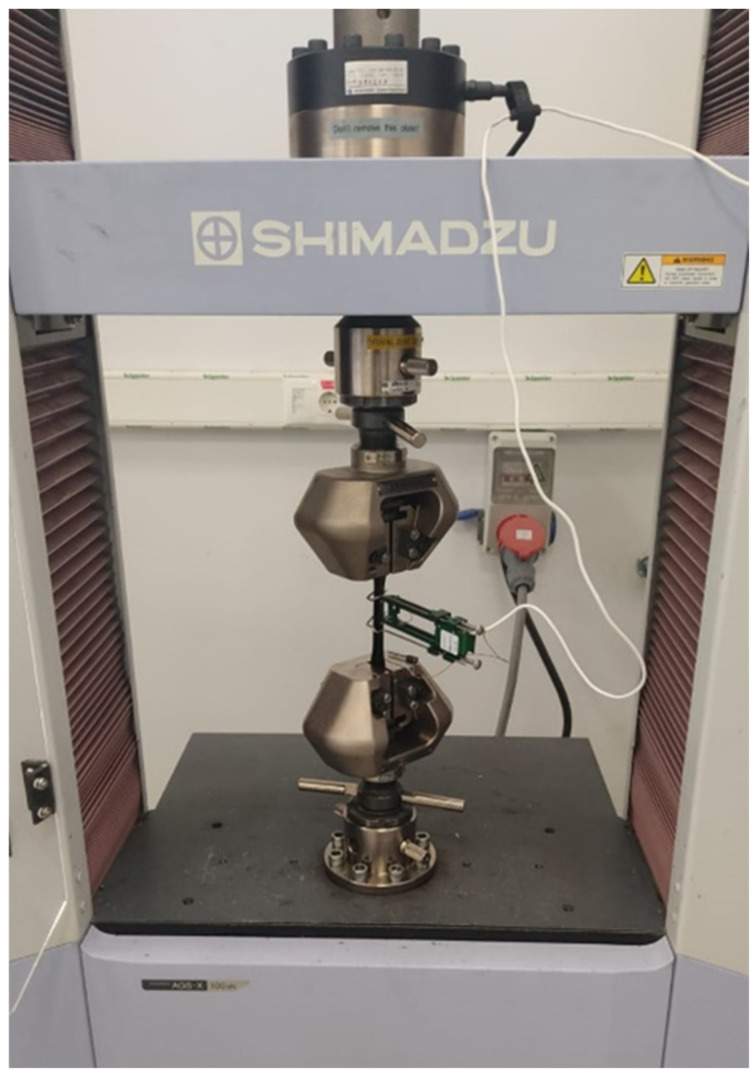
Test setup with specimen.

The mechanical parameters, including the modulus, tensile deformation, and tensile strength, were assessed by ASTM D638 [30] using universal testing equipment (Shimadzu 10 kN, Shimadzu AG-X model, Kyoto, Japan) (Figure 15). The experiments were conducted at room temperature with a constant displacement speed of 2 mm/min. The displacement of the specimens was measured using an extensometer. Stress vs. strain data were acquired and compared with neat epoxy resin and between the samples (Figure 16).

**Figure 17:**
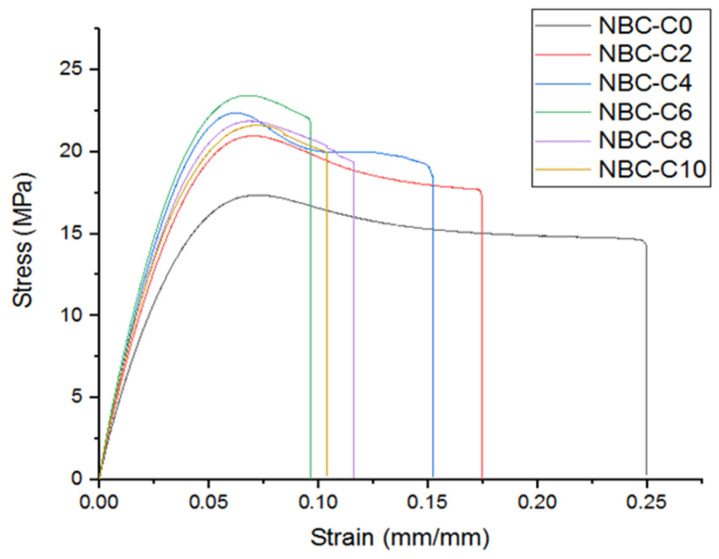
Stress–strain curve comparison.

The tensile test results were analyzed to evaluate the mechanical performance of the composites containing different weights (wt.%) of nano-BC, as shown in Figure 17. According to the obtained stress–strain curves, it was observed that the stress along the elastic region increased linearly with the strain in all samples and then transitioned to the plastic deformation region, at which point the breaking point was reached. With the increase in the nano-BC content, a significant increase in the modulus of elasticity was observed, while in terms of the maximum tensile strength, it was seen that the highest values were reached in the 6 wt.% range. However, at 10 wt.%, it was determined that the mechanical strength decreased, and the material became more brittle when the composites had a higher wt.% of nano-BC. It was seen that the nano-BC content could weaken the bonding within the matrix and reduce the ductility of the material after a certain level.

**Figure 18:**
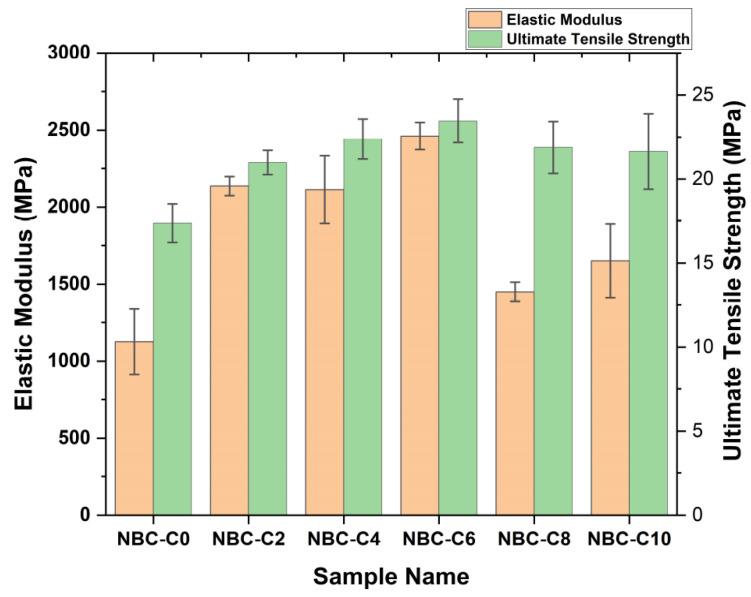
Ultimate tensile strength (UTS) and elastic modulus (E) of nano-BC composites.

In addition, Figure 18 gives the ultimate tensile strength (UTS) results comparatively for all the samples. In the pure epoxy matrix (0 wt.% C), the UTS was measured as 17.37 MPa, while this increased significantly with the addition of the nano-BC. With the nano-BC contents at 2 wt.% and 4 wt.%, the UTS increased to 20.98 MPa and 22.38 MPa, respectively, with the highest strength being obtained at 6 wt.%, which had a UTS value of 23.46 MPa. However, the UTS decreased to 21.88 MPa and 21.64 MPa in the samples with 8 wt.% and 10 wt.% nano-BC contents, respectively. These results show that a high nano-BC content can adversely affect the homogeneous distribution in the epoxy matrix, creating internal stresses and increasing the material’s brittleness.

The presence of oxygen-containing functional groups on the surface of the biochar increased the thermal stability of the polymer composites by facilitating the formation of a stable char layer during thermal degradation and preventing the spread of oxidation reactions. Furthermore, in the composites containing biochar, the facilitated stress transfer between the filler and the matrix led to an increase in the mechanical performance and also limited the mobility of the polymer chains, resulting in the formation of a denser cross-linked network in the epoxy matrix and the strengthening of the structural strength [12,35]. Also, it has been reported that the surface area of biochar can be altered by pyrolytic process parameters and is directly related to its morphological properties. The changes made in this regard have demonstrated the potential to elevate the mechanical and thermal stability balance of these composites to the desired level [24].

### 3.11. Static Toughness

**Figure 19:**
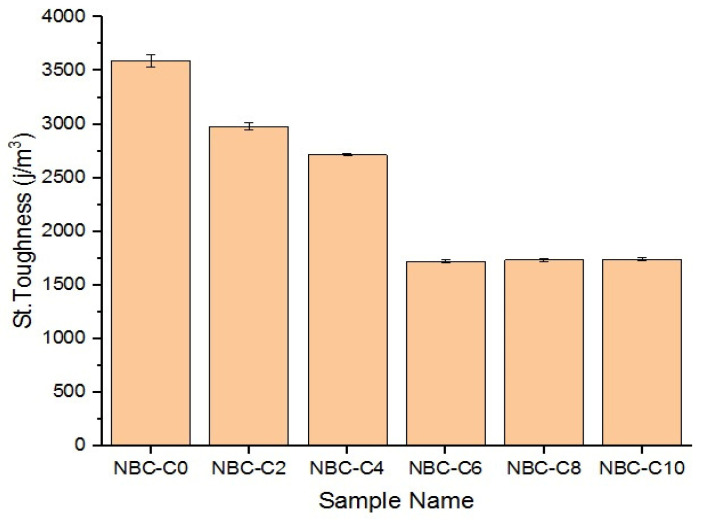
Static toughness of nano-BC composites.

Static toughness is a measure of the maximum capacity of a material to withstand load before breakage. Figure 19 shows the static toughness values of the polymer composites containing different percentages of nano-BC by weight. The neat epoxy specimen (0 wt.% C) had the highest static toughness (3.592 MJ/m^3^). As the content of nano-BC increased, the toughness decreased significantly. The static toughness in the 2 wt.% and 4 wt.% nano-BC samples was calculated as 2.980 MJ/m^3^ and 2.8915 MJ/m^3^, respectively. The lowest static toughness was observed in the sample containing 6 wt.% nano-BC (1.717 MJ/m^3^). A small recovery was observed in the sample containing 8 wt.% nano-BC, and a value of 1.346 MJ/m^3^ was obtained. The sample containing 10 wt.% nano-BC had a similar static toughness (1.765 MJ/m^3^) as the 6 wt.% nano-BC sample, but the increase was quite low. According to these results, the 0 wt.% nano-BC sample exhibited ductile behavior and absorbed more energy until it was broken. The material became brittle in the sample with the lowest toughness, 6 wt.% nano-BC, and it had a decreased energy absorption capacity.

## 4. Conclusions

In this study, nano-biochar (nano-BC) obtained from olive pulp, a sustainable and environmentally friendly source, made significant improvements to the mechanical and thermal properties of epoxy-based thermoset resins.

Characterization of the nano-BC revealed a specific surface area of 39.86 m^2^/g, an average particle diameter of 225.5 nm, and the low polydispersity index. SEM and EDS analyses revealed a layered, carbon-rich, and oxygen-containing functional-group-containing structure; XRD and Raman analyses confirmed the semi-crystalline graphitic character of the material. This structure created strong interfacial interactions with the epoxy matrix, thus providing advantages in terms of charge transfer and the thermal barrier effect.

In mechanical tests, with a 6% additive ratio, the ultimate tensile strength increased by 35%, alongside increases in the elastic modulus and the Shore D hardness. DMA analyses revealed that the sample with 6% additive showed the highest storage modulus (E′). In addition, the softening onset temperature (Tonset) increased as the additive ratio increased up to 6%, proving that the thermomechanical stability increased. With the addition of 6 wt.% nano-BC, the storage elastic modulus decreased as the polymer network, which was saturated mainly in terms of structure additives, was disrupted and weakened and could not transfer the stress onto the particle structure with a lower strength than the matrix, and this became more pronounced with the increase in temperature.

The temperature at which the deterioration began was lowered when the addition rate of the nano-BC was increased to eight weight percent, but the temperature at which the degradation rate peaked was raised. By serving as a heat barrier, the increased nano-BC addition postponed the breakdown of the matrix’s primary structure and decreased the separation of volatile chemicals from the structure.

However, there were limitations observed in this study when the nano-BC loading exceeded 6%. Increasing the addition of the nano-BC (8% and 10%) led to a consolidation effect (the amount of matrix to be deformed decreased, leading to the degradation of the polymer network), resulting in a decreased tensile strength, elastic modulus, and toughness (stress bending area). The results indicated that the filler phase has the potential to limit ductile behavior and make the structure brittle.

The mechanism involves the transfer of matrix mechanical effects onto the nano-BS and the interaction between the nano-BC particles and the matrix interface. However, it is predictable that when nano-BC of the size indicated in this study (~225 nm) is added at doping ratios higher than 6% by mass, the tensile strength will decrease due to the weakening and degradation of the polymer network.

In conclusion, this study showed that nano-BC is a highly effective reinforcement element for epoxy matrix systems at low additive ratios. Although the nano-sized carbon-based structure provides both mechanical strength and thermal stability, it can negatively affect the integrity of the structure when a certain additive threshold is exceeded. The findings show that biochar-based filling materials can be efficiently used to produce economically efficient, environmentally friendly, and high-performance composites. In particular, the processing of agricultural wastes such as olive pulp into advanced functional engineering materials is considered an important contribution to both the circular economy and material science.

## Figures and Tables

**Table 1:** Composite sample mixture content.

Sample	Resin (gram)	Hardener (gram)	Nano-BC Filler (gram)
NBC-C0 (Control Sample)	50	25	0
NBC-C2	49	24.5	1.5
NBC-C4	48	24	3
NBC-C6	47	23.5	4.5
NBC-C8	46	23	6
NBC-C10	45	22.5	7.5

**Table 2:** Nano-BC particle elemental distribution (wt.%).

Map Sum Spectrum				
Element	Line Type	Weight%	Weight% Sigma	Atomic%
C	K series	81.23	0.26	86.16
O	K series	16.36	0.26	13.02
K	K series	1.50	0.03	0.49
Ca	K series	0.72	0.03	0.23
Si	K series	0.12	0.01	0.05
Mg	K series	0.08	0.02	0.04
Total		100.00		100.00

**Table 3:** Nano-BC particle size measurement results.

Measurement No.	Ave. Diameter (nm)	Polydispersity Index	Mean. (nm)	D-10% (nm)	D-50% (nm)	D-90% (nm)
1	225.7	0.174	194.6	159.6	185.4	216.7
2	247.3	0.2	154.6	128	147.2	171.2
3	225.1	0.176	209.4	169.7	199.3	234.7
4	206.3	0.196	190.2	155.4	181.3	212.2
5	223.1	0.169	194.6	159.9	185.4	216.5
Average	225.5	0.183	188.68	154.52	179.72	210.26

## Data Availability

The original contributions presented in the study are included in the article; further inquiries can be directed to the corresponding author.
